# Loneliness patterns across time and subsequent risk of psychotic experiences, depression, anxiety, and diminished well-being in adolescents

**DOI:** 10.1111/jcpp.70114

**Published:** 2026-01-22

**Authors:** Zui C Narita, Jordan DeVylder, Gemma Knowles, Syudo Yamasaki, Mitsuhiro Miyashita, Daniel Stanyon, Satoshi Yamaguchi, Tomohiro Shinozaki, Ryo Sasaki, Rui Zhou, Shuntaro Ando, Craig Morgan, Paola Dazzan, Toshiaki A Furukawa, Kiyoto Kasai, Ian Kelleher, Atsushi Nishida

**Affiliations:** 1 Department of Behavioral Medicine, National Institute of Mental Health, National Center of Neurology and Psychiatry, Tokyo, Japan; 2 Silver School of Social Work, New York University, New York, New York City, USA; 3 Unit for Mental Health Promotion, Research Center for Social Science & Medicine, Tokyo Metropolitan Institute of Medical Science, Tokyo, Japan; 4 Economic and Social Research Council (ESRC) Center for Society and Mental Health and Health Service and Population Research Department, Institute of Psychiatry, Psychology, and Neuroscience, King’s College London, London, UK; 5 Interfaculty Initiative in Information Studies, the University of Tokyo Department of Biostatistics, School of Public Health, Graduate School of Medicine, the University of Tokyo; 6 Department of Neuropsychiatry, Graduate School of Medicine, The University of Tokyo, Tokyo, Japan; 7 Division of Psychiatry, Centre for Clinical Brain Sciences, University of Edinburgh, Edinburgh, UK; 8 School of Medicine, University College Dublin, Dublin, Ireland; 9 School of Medicine, University of Oulu, Oulu, Finland; 10 St. John of God Hospitaller Services Group, Hospitaller House, Stillorgan, Dublin, Ireland; 11 Department of Psychological Medicine, Institute of Psychiatry, Psychology, and Neuroscience, King’s College London; 12 Department of Health Promotion and Human Behavior, Kyoto University Graduate School of Medicine/ School of Public Health, Kyoto, Japan; 13 The International Research Center for Neurointelligence (WPI-IRCN), University of Tokyo Institutes of Advanced Study (UTIAS), Tokyo, Japan

**Keywords:** Major depressive disorder (MDD), schizophrenia, psychosis, anxiety, adolescence

## Abstract

**Background:**

Evidence on the association between loneliness and psychotic experiences in adolescents remains limited. Moreover, loneliness has typically been assessed at a single time point, which fails to capture its dynamic nature. We hypothesized that persistent loneliness, assessed across repeated measures, would be associated with psychotic experiences and other mental health problems.

**Methods:**

Using longitudinal data from 3,171 participants in the Tokyo Teen Cohort, we applied the g-formula. We analyzed how loneliness patterns at ages 12 and 14 were associated with psychotic experiences, depression, anxiety, and diminished well-being at age 16, accounting for time-fixed and time-varying confounders. Missing data were handled using multiple imputation by chained equations.

**Results:**

Persistent loneliness was associated with increased risk and greater severity of psychotic experiences (RD 7.1%, 95% CI 0.8% to 14.3%; RR 2.44, 95% CI 1.16 to 4.11; *β* 0.28, 95% CI 0.10 to 0.48). Incident loneliness at age 14 showed similar associations. No association was found for adolescents whose loneliness had remitted by age 14 (RD -1.3%, 95% CI -3.6% to 1.2%; RR 0.73, 95% CI 0.31 to 1.26; *β* 0.01, 95% CI -0.04 to 0.08). Sensitivity analyses using marginal structural models yielded results that were largely unchanged. Findings were generally similar for other mental health problems. Associations were consistent across genders, although the association with well-being appeared particularly important for girls.

**Conclusions:**

The dynamics of loneliness are associated with a wide range of mental health problems in adolescents. The risk may not be permanent and could be mitigated if loneliness remits. Further research examining interventions that target loneliness is warranted.

## Introduction

Loneliness is a distressing subjective experience of inadequate social connection (Gayer-Anderson & Morgan, 2013; Hawkley & Cacioppo, 2010). Adolescents’ feelings of loneliness have increased sharply over the past 15 years, a trend that has been linked to recent changes in social interaction patterns (Twenge, Spitzberg, & Campbell, 2019). Adolescence represents a developmental period characterized by shifts in social relationships, highlighting the importance of understanding how loneliness during this life stage affects mental health outcomes (Twenge et al., 2019).

Previous studies have found associations between loneliness and psychotic experiences (Bell et al., 2023; Narita et al., 2021; Narita, DeVylder, et al., 2024; Narita, Stickley, & DeVylder, 2020; van der Werf, van Winkel, van Boxtel, & van Os, 2010). Emerging longitudinal evidence has begun to clarify this link. Cross-lagged analyses showed that loneliness was associated with psychotic experiences in undergraduates (Tan, Barkus, & Favelle, 2021). One cohort study indicated that psychotic experiences at age 12 predicted loneliness at age 14, suggesting that psychotic experiences may not simply be a consequence of loneliness but could instead precede and contribute to subsequent loneliness (Endo et al., 2022). Another cohort study found that adolescents with heightened or increasing loneliness were more likely to be prescribed antipsychotics in adulthood (Rodríguez-Cano, Lotre, von Soest, Rognli, & Bramness, 2024). The European Gene–Environment Interactions in Schizophrenia study showed that prolonged childhood and adolescent loneliness was associated with elevated odds of schizophrenia-spectrum disorders (Andreu-Bernabeu et al., 2025). Potential mechanisms to explain the association might include the induction of maladaptive cognition (Hawkley & Cacioppo, 2010). Also, social deafferentation (Hoffman, 2007; Selten & Ormel, 2023), by reducing social input to neural systems, may trigger the generation of spurious social meaning, such as delusions. Social defeat (Bielawski, Rejek, & Misiak, 2025; Cosse et al., 2025), such as humiliation, may heighten dopaminergic sensitivity and lead to psychotic experiences. Despite this existing body of research, loneliness has typically been assessed at a single time point, even in longitudinal studies. Although cross-lagged panel models have been used and involve repeated assessments of loneliness (Tan et al., 2021), they are not designed to address time-varying exposure and confounding, which may restrict their utility for causal inference.

To properly assess the evolving impacts of loneliness patterns across time on mental health, it is necessary to model loneliness as a time-varying exposure. In reality, loneliness is a time-varying experience that can fluctuate with life circumstances (Endo et al., 2022). Analyzing time-varying exposures introduces methodological challenges, including time-varying confounding and exposure-confounder feedback, where prior values of the exposure influence future confounders, which then affect the subsequent exposure. Traditional outcome regression cannot appropriately account for these dynamics. Thus, addressing the causal inference of loneliness patterns across time requires analytical methods designed to handle such complexities.

To address these limitations, we investigated the impact of repeatedly assessed loneliness on adolescent mental health using data from the Tokyo Teen Cohort (TTC), a longitudinal study of 3,171 participants. We applied g-methods (the g-formula and marginal structural models) to examine the association between loneliness patterns across time and subsequent psychotic experiences among adolescents, accounting for both time-fixed and time-varying confounding. Given that empirical evidence on time-varying loneliness in adolescence remains limited, we drew on emerging developmental findings suggesting that different temporal patterns of loneliness may have distinct implications. In particular, a recent longitudinal study found that adolescents who experienced loneliness only in early adolescence generally had better mental health outcomes in late adolescence than those with persistent or later-emerging loneliness (Matthews et al., 2023). While this study did not evaluate psychotic experiences, these findings highlight the potential importance of distinguishing between persistent, incident, and remitting loneliness patterns. We hypothesized that persistent loneliness across multiple waves would be associated with increased risk and severity of subsequent psychotic experiences, whereas loneliness that remitted between assessments would show attenuated associations. We also investigated the association between loneliness and other important mental health problems, namely depression, anxiety, and diminished well-being, given the past report (Loades et al., 2020) and the alignment with theoretical models, such as the social deafferentation hypothesis (Hoffman, 2007; Selten & Ormel, 2023). Although psychotic experiences were a central focus of our research question, our aim was to provide a broader understanding of the mental health consequences of adolescent loneliness.

## Methods

### Participants

The TTC is based on the Tokyo Early Adolescence Survey (T-EAS). As previously described (Ando et al., 2019), all children listed in the resident registers of three municipalities in metropolitan Tokyo (Setagaya, Mitaka, and Chofu) who were born between September 2002 and August 2004 (N = 18,830) were identified as the sampling frame. Owing to a limited research budget, 14,553 children were randomly selected from this frame for invitation to the T-EAS. Among these, 10,234 children were reachable, and 4,478 agreed to participate at age 10. For the subsequent establishment of the TTC, the study team planned to recruit approximately 3,000 participants for longitudinal follow-up (Ando et al., 2019). To achieve this target while ensuring adequate representation of lower-income households, all 620 T-EAS participants whose annual household income was below 5,000,000 JPY were invited to join the cohort. An additional 2,551 children were then randomly selected from the remaining 3,858 T-EAS participants, resulting in 3,171 children being invited to the TTC. These 3,171 children constitute the baseline sample for the present study (see Figure S1). Data were obtained at four time points when individuals were at ages 10 (November 2012 to January 2015), 12 (August 2014 to January 2017, follow-up rate 94.8%), 14 (August 2014 to January 2017, follow-up rate 84.1%), and 16 (February 2019 to September 2021, follow-up rate 82.5%). Trained interviewers administered self-report questionnaires to child-parent pairs at each time point. As the TTC design determined the recruitment target, the final sample size of 3,171 was based on feasibility and fieldwork capacity rather than a formal a priori statistical power calculation.

All invited children were included in the present analyses, with missing data handled using multiple imputation. The Institutional Review Boards of the Tokyo Metropolitan Institute of Medical Science (12-35), the University of Tokyo (10057), and SOKENDAI (2012002) approved the present study. All parents gave written informed consent.

### Loneliness (exposure)

Loneliness at ages 12 and 14 was evaluated using an item (“I felt lonely”) from the Short Mood and Feelings Questionnaire (SMFQ), a standard 13-item self-report measure (Angold, Costello, Messer, & Pickles, 1995; Sharp, Goodyer, & Croudace, 2006). The item had three response options: “always,” “sometimes,” and “never.” We created a dichotomous variable (never vs. sometimes or always). A separate multi-item or continuous measure of loneliness was not available in the TTC dataset. We used a dichotomous specification in this manner because marginal structural models are known to perform most reliably with binary exposures (Hernán & Robins, 2020), and also because the number of participants in the ‘always’ category was limited, which could further limit the sample size and the number of cases in the persistent loneliness group.

### Mental health problems (outcomes)

We evaluated psychotic experiences (Narita, 2025) at ages 10 and 16 using the Adolescent Psychotic-like Symptom Screener (APSS), an established seven-item self-report questionnaire (Kelleher, Harley, Murtagh, & Cannon, 2011; Laurens et al., 2007). The questionnaire had three response options: “yes, definitely,” “maybe,” and “no, never,” scored with 1, 0.5, and 0 points, respectively. Items were summed into a total score (possible range 0–7); a higher score suggests a higher degree of psychotic experiences. Details of items can be found in Table S1. Our data showed acceptable internal consistency (Cronbach’s *α*, 0.71). Psychotic experiences at age 12 were evaluated via items from the schizophrenia section of the Diagnostic Interview Schedule for Children (Costello, Edelbrock, & Costello, 1985). For the analysis using the dichotomous outcome, selecting “Yes, definitely” on at least one item of the APSS constituted the presence of psychotic experiences (Kelleher et al., 2011).

To evaluate depressive symptoms at ages 10, 12, and 16, we used the SMFQ (Angold et al., 1995; Sharp et al., 2006). The questionnaire had three response options: “always,” “sometimes,” and “never,” scored with 2, 1, and 0 points, respectively. Items were summed into a total score (possible range 0–26); a higher score suggests a higher degree of depressive symptoms. Our data showed strong internal consistency (Cronbach’s *α*, 0.92). For the analysis using the dichotomous outcome, a score of ≥ 8 on the SMFQ constituted the presence of depression (Angold et al., 1995). Because the SMFQ contains an item assessing loneliness, we excluded this item when constructing the depressive symptoms score used in the main analyses. Results based on the full SMFQ that includes the loneliness item are reported as a sensitivity analysis.

To evaluate anxiety at ages 10, 12, and 16, we used the Child Behavior Checklist (CBCL) (Itani, 2001), a widely used caregiver-reported questionnaire consisting of 118 items. To mitigate the burden on participants, we selected 84 items from the CBCL for our study. Of these, 14 items were specifically designed to evaluate anxiety, derived from the CBCL anxiety scale, which initially consisted of 16 items (Iijima et al., 2019). Each item was assessed with three response options: “not true,” “somewhat or sometimes true,” and “very true or often true.” A total score was obtained by summing the responses for each item, with higher scores indicating a higher degree of anxiety symptoms (possible range 0–28). Our data showed acceptable internal consistency (Cronbach’s *α*, 0.78). For the analysis using the dichotomous outcome, a T-score of ≥ 65 constituted the presence of anxiety (Funabiki & Murai, 2017).

To evaluate well-being at ages 10, 12, and 16, we employed the World Health Organization-Five Well-Being Index (WHO-5), a standard five-item self-report questionnaire (Topp, Østergaard, Søndergaard, & Bech, 2015). The WHO-5 evaluated positive aspects of mental health, such as mood, vitality, and general interest in life. Each questionnaire had six response options: “not at all,” “a little,” “somewhat,” “quite a bit,” “a lot,” and “all of the time.” A total score was obtained by summing the responses and multiplying by four (possible range 0–100), with higher scores indicating better well-being. Our data demonstrated good internal consistency (Cronbach’s α, 0.81). For the analysis using the dichotomous outcome, a score of ≤ 50 on the WHO-5 constituted the diminished well-being (Topp et al., 2015)

### Covariates

We accounted for confounders based on the modified disjunctive cause criterion to adjust for potential causes of the exposure, outcome, or both, excluding instrumental variables and including covariates that served as proxies for unmeasured variables that are common causes of both the exposure and the outcome (VanderWeele, 2019). The model included age, gender, IQ (Koenen et al., 2009)^,^ body mass index (Perry et al., 2021), household income (Sareen, Afifi, McMillan, & Asmundson, 2011), physical punishment (Edwards, Holden, Felitti, & Anda, 2003), living arrangement (Narita et al., 2024), neighborhood cohesion (Fedina et al., 2021; Narita, Knowles, et al., 2020), problematic internet use (Narita, Ando, et al., 2024), and loneliness. Details on covariates are shown in Method S1. Per analysis, we included the baseline level of each mental health condition. Furthermore, we accounted for body mass index, physical punishment, problematic internet use, and the level of each mental health condition at age 12 as time-varying confounders, which may be affected by loneliness at age 12 and may also confound the association between loneliness at age 14 and mental health problems at age 16. Figure S2 illustrates the hypothesized associations between loneliness (exposures at ages 12 and 14), covariates (confounders at ages 10 and 12), and mental health problems (outcomes at age 16).

### Statistical analysis

All analyses were conducted using R-4.5.0. First, we analyzed the association of time-varying loneliness at ages 12 and 14 with the severity of mental health problems at age 16. We applied the parametric g-formula (Hernán & Robins, 2020), adjusting for the baseline and time-varying confounders at ages 10 and 12. In short, we simulated counterfactual time-varying confounders and outcomes that would have been observed if everyone had been allocated to a specific pattern of loneliness, namely: (1) never lonely at both ages 12 and 14, (2) lonely at age 12 but not lonely at age 14, (3) not lonely at age 12 but lonely at age 14, and (4) lonely at both ages 12 and 14. We fitted confounder models at age 12, including loneliness at age 12 and confounders at age 10. We then fitted outcome models at age 16, including the interaction term between loneliness at ages 12 and 14 and confounders at ages 10 and 12. We computed the differences in average severity scores using these counterfactual simulated outcomes, thereby showing how severity differed across four patterns of loneliness. Analyses were also stratified by gender (Liu, Zhang, Yang, & Yu, 2020; Narita, Knowles, Yamasaki, Kasai, & Nishida, 2025).

To examine the association between loneliness and the presence of mental health problems, we conducted analyses using dichotomized outcomes. We obtained the risk difference (RD) and risk ratio (RR) from the g-formula simulating counterfactual probabilities under four patterns of loneliness. We evaluated the robustness of the estimates to unmeasured confounding by analyzing E-values for the RR (VanderWeele & Ding, 2017). Analyses were also stratified by gender (Liu et al., 2020; Narita, Knowles, et al., 2025).

Missing data were handled via multiple imputation by chained equations, and estimates were synthesized across 20 imputed datasets. Confidence intervals (CIs) were calculated using a percentile bootstrap approach known as MI Boot with pooled samples, where we drew 200 bootstrap samples from each of the 20 imputed datasets, yielding a total of 4,000 estimates (Schomaker & Heumann, 2018).

We conducted five sensitivity analyses. First, we used marginal structural models (Shinozaki & Suzuki, 2020) to examine severity (a continuous outcome). We fitted propensity models to calculate inverse probability weights for loneliness at ages 12 and 14 using separate logistic models for exposure at age 12 (including confounders at age 10) and at age 14 (including exposure at age 12 and confounders at ages 10 and 12). The weights were multiplied into a single weight per person and used for fitting saturated marginal structural models with the interaction term between loneliness at ages 12 and 14. For each imputed dataset, point estimates and robust (sandwich) variances were obtained; CIs were computed using Rubin’s rules. Second, we applied a similar marginal structural model approach to dichotomous outcomes. Here, we obtained marginal odds ratios (ORs) for comparison with other literature. Third, we further adjusted for time spent gaming (< 1 hour/day vs. ≥ 1 hour/day), considering its potential importance as a confounder (Narita, DeVylder, et al., 2025). Fourth, to assess depressive symptoms, we used the full SMFQ that includes the loneliness item. Fifth, we examined loneliness patterns at ages 12 and 16. Because the mental health outcomes were evaluated at age 16, incorporating loneliness at age 16 in the analysis could introduce the risk of reverse causation; nonetheless, we included it as a sensitivity analysis to contextualize the findings.

This study did not have a pre-registered analysis plan.

## Results

### Baseline characteristics

The baseline characteristics of the 3,171 study participants are shown in Table 1. Adolescents who experienced loneliness at age 12 were more likely to be female (55.6%) compared with those who did not report loneliness (45.4%). At age 10, they also reported higher levels of problematic internet use, were more likely to experience loneliness, had higher depressive symptoms and anxiety symptoms, and lower levels of well-being. Other characteristics did not substantially differ across groups. Details of missing data are summarized in Table S2.

### Loneliness patterns across time and subsequent severity of mental health problems

**Figure 1:**
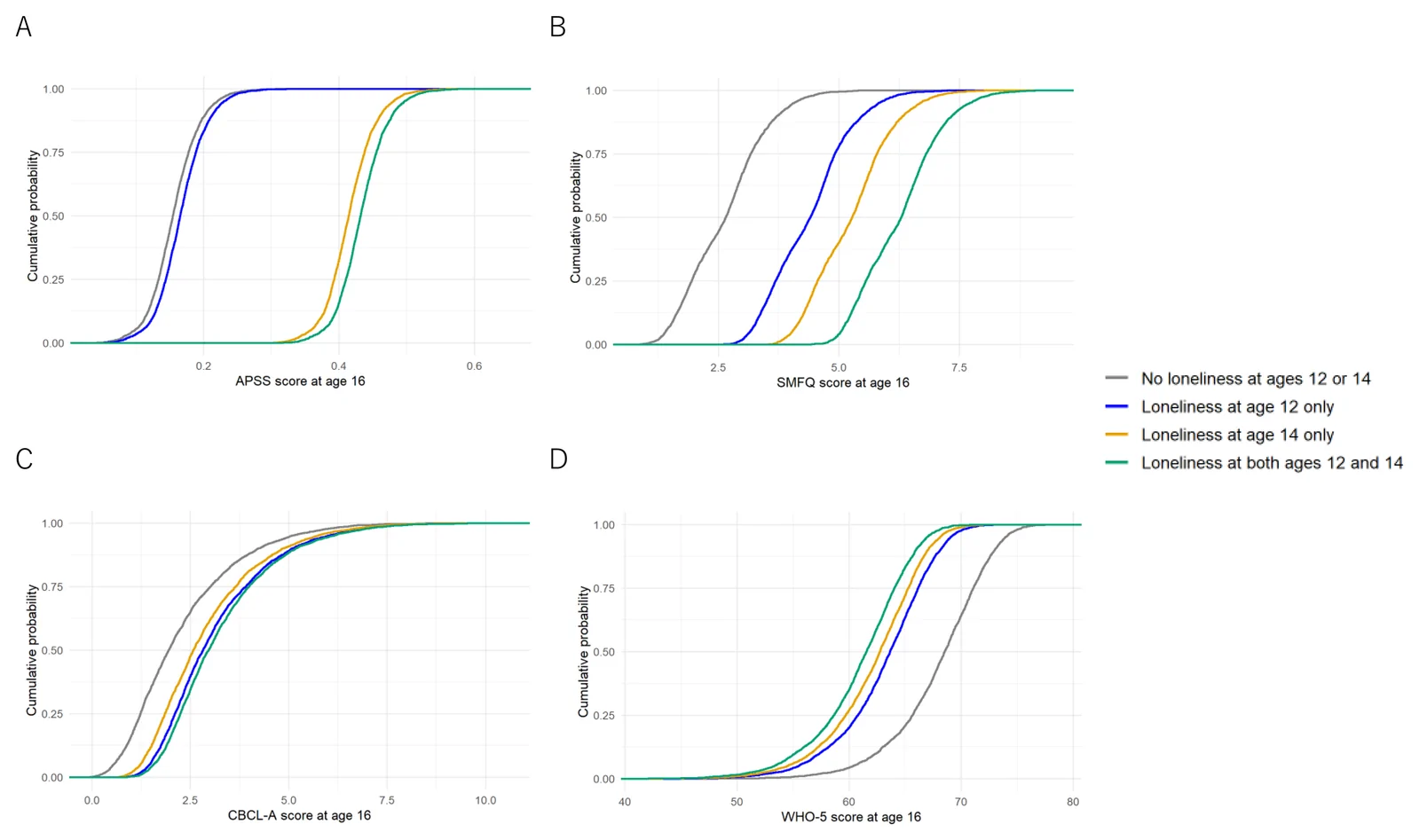
Cumulative distribution of mental health outcomes at age 16 by loneliness status at ages 12 and 14: (A) Psychotic experiences score (APSS), (B) Depressive symptoms score (SMFQ), (C) Anxiety symptoms score (CBCL-A), and (D) Well-being score (WHO-5). APSS, Adolescent Psychotic-Like Symptom Screener; SMFQ, Short Mood and Feelings Questionnaire; CBCL-A, Child Behavior Checklist–Anxiety Scale; WHO-5, World Health Organization-Five Well-Being Index

The prevalence of loneliness was 14.6% at age 12 and 9.8% at age 14. Figure 1 and Table 2 show how loneliness patterns across time were associated with the severity of mental health problems at age 16. Compared with adolescents who were never lonely, those with persistent loneliness at ages 12 and 14 had a higher mean score of psychotic experiences (*β* 0.28, 95% CI 0.10 to 0.48), depressive symptoms (*β* 3.62, 95% CI 2.06 to 5.07), anxiety symptoms (*β* 1.00, 95% CI 0.25 to 1.78), and a lower mean score of well-being (*β* -7.01, 95% CI -12.12 to -1.68). Incident loneliness at age 14 showed largely similar associations. In adolescents for whom loneliness had remitted by age 14 (lonely at age 12 only), the association with psychotic experiences was not observed (*β* 0.01, 95% CI -0.04 to 0.08), and associations with other mental health problems were weaker. These findings were largely similar across genders, although girls showed larger absolute estimates for well-being.

### Loneliness patterns across time and subsequent risk of mental health problems

**Figure 2:**
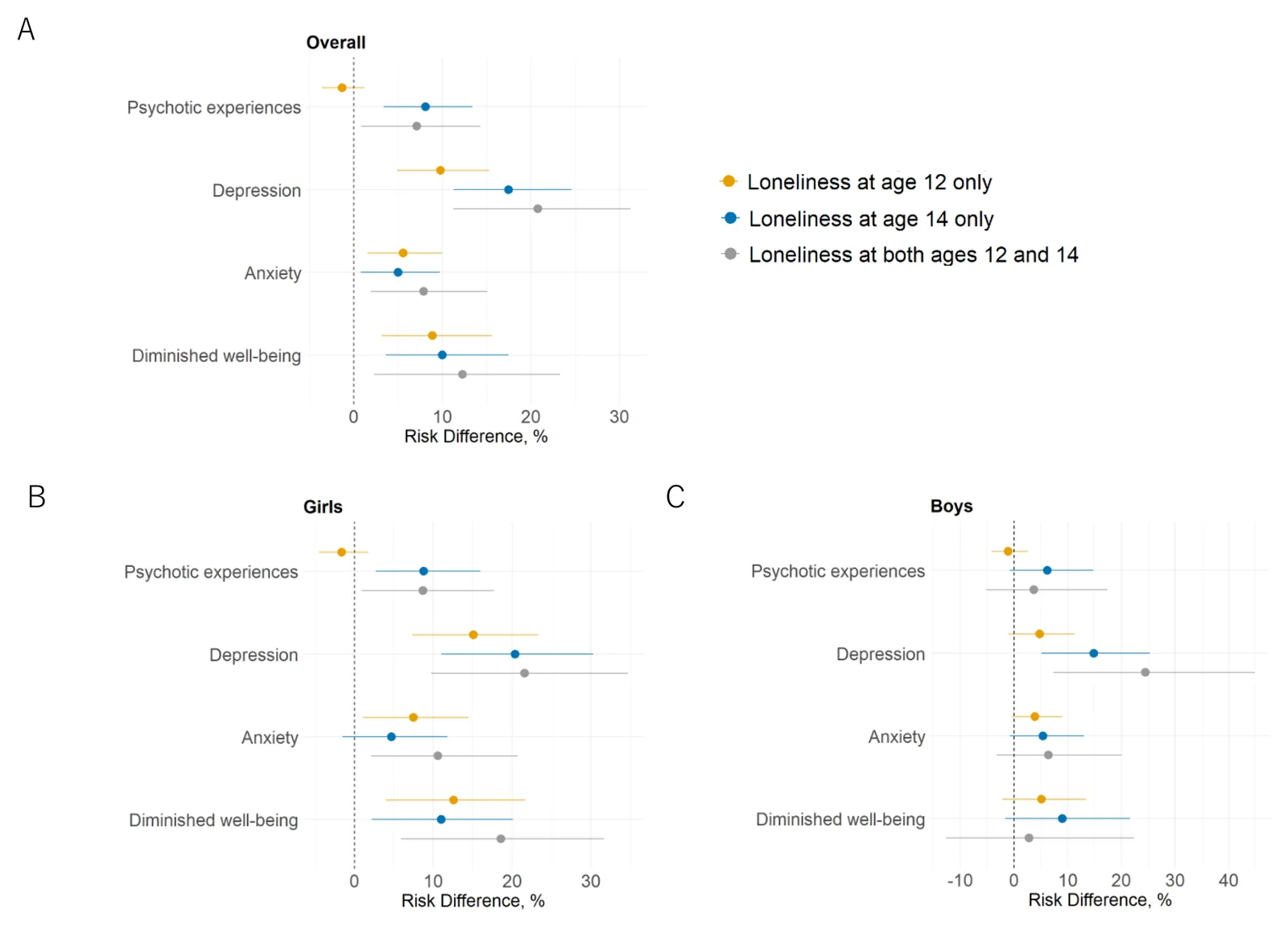
Loneliness patterns (ages 12 and 14) and subsequent risk of mental health problems (age 16) evaluated by the g-formula: Risk difference in (A) Overall sample, (B) Girls, and (C) Boys.

Next, we analyzed the association between loneliness patterns across time and the presence of mental health problems (Figures 1 and 2, and Table S3). Compared with adolescents who were never lonely, those with persistent loneliness at ages 12 and 14 had an increased risk of psychotic experiences (RD 7.1%, 95% CI 0.8% to 14.3%; RR 2.44, 95% CI 1.16 to 4.11) depression (RD 20.8%, 95% CI 11.2% to 31.3%; RR 2.96, 95% CI 2.01 to 4.05), anxiety (RD 7.9%, 95% CI 1.9% to 15.1%; RR 2.22, 95% CI 1.27 to 3.46) and diminished well-being (RD 12.3%, 95% CI 2.3% to 23.3%; RR 1.67, 95% CI 1.12 to 2.29) at age 16. Incident loneliness at age 14 showed largely similar associations. For adolescents who were lonely only at age 12, again the association with psychotic experiences was not observed (RD -1.3%, 95% CI -3.6% to 1.2%; RR 0.73, 95% CI 0.31 to 1.26). Associations with other mental health problems were weaker. E-values showed that some observed associations between time-varying loneliness and the incidence of mental health problems were reasonably robust to unmeasured confounders (Table S4). These findings were largely similar across genders, although girls showed larger RDs for diminished well-being.

### Sensitivity analyses

First, we used marginal structural models to evaluate severity, yielding similar estimates (Table S5). Second, we used marginal structural models to examine dichotomous outcomes and found increased ORs that are consistent with the main findings (Table S6). Third, we further adjusted for time spent gaming at ages 10 and 12, and found that the estimates remained similar (Table S7). Fourth, for depressive symptoms, the findings were similar when using the full SMFQ that includes the loneliness item (Table S8). Fifth, the results were largely unchanged when examining loneliness patterns at ages 12 and 16, although the absolute values of the estimates were generally larger (Table S9).

## Discussion

In a large population-based cohort of youth, we found that persistent loneliness was associated with increased risk and severity of psychotic experiences and other mental health problems. Importantly, however, we found that the risk and severity ameliorated when loneliness remitted between ages 12 and 14, suggesting that the negative impact of loneliness on adolescent mental health is not permanent and could be mitigated. This was particularly evident for psychotic experiences, where the risk and severity were no longer elevated. The findings were largely consistent across genders, although the association with well-being appeared particularly important for girls. Our findings remained robust across five sensitivity analyses.

Our findings support previous research demonstrating a relationship between loneliness and psychotic experiences (Andreu-Bernabeu et al., 2025; Bell et al., 2023; Narita, DeVylder, et al., 2024; Narita et al., 2021; Narita, Stickley, et al., 2020; Rodríguez-Cano et al., 2024; Tan et al., 2021; van der Werf et al., 2010). This study is the first, however, to apply robust causal methods to observational data to examine whether remission of loneliness in adolescents is associated with a similar reduction in the risk and severity of psychotic experiences. While our observational data cannot definitively prove a causal relationship between the reduction in loneliness and the reduction in psychotic experiences, our rigorous causal inference methods provide important support for the possibility of loneliness as a treatment target to reduce the risk and severity of psychotic experiences in developing adolescents.

Interestingly, adolescents who experienced loneliness at age 14 only showed associations with mental health outcomes that were comparable to those observed for adolescents with persistent loneliness. These findings align with prior research (Matthews et al., 2023) and suggest that the associations may not reflect a simple dose–response relationship. One interpretation is that mid-adolescence may be a period during which loneliness is more closely associated with later mental health outcomes, regardless of whether loneliness occurred earlier. Another possibility is that loneliness occurring closer in time to the outcome assessment may have shown a stronger association with the outcomes. Taken together, these observations underscore that later-emerging loneliness may be as relevant as persistent loneliness when considering subsequent mental health problems.

Further research will be necessary to identify the mechanisms underlying the relationship between remission of loneliness and reduced risk and severity of psychotic experiences via methods like mediation analysis (Narita, Miyashita, Furukawa, & Nishida, 2025; VanderWeele, 2015). Although attenuation of the associations was observed across all outcomes when loneliness remitted, this attenuation appeared particularly marked for psychotic experiences, indicating a degree of outcome specificity. The process of reconnecting with peers may directly counteract the maladaptive cognitions (Hawkley & Cacioppo, 2010) that develop during periods of isolation. Re-establishing social relationships can reverse social deafferentation (Hoffman, 2007; Selten & Ormel, 2023) by providing essential social feedback, helping adolescents correct unusual or paranoid thoughts. Social interaction might reduce adolescents’ vigilance for social threats, thus alleviating a major source of stress and further mitigating deleterious thought patterns. Psychotic experiences were associated with incident loneliness at age 14 to a similar degree as with persistent loneliness, which may be partly explained by the social defeat hypothesis (Bielawski et al., 2025; Cosse et al., 2025). Acute experiences of exclusion during socially sensitive periods like mid-adolescence may be perceived as humiliating, which could help explain why timing matters for psychotic experiences. Taken together, the mechanisms reviewed here may be more specific to psychotic experiences and help explain why changes in loneliness are most distinctly reflected in this domain, while they may also be somewhat relevant to other mental health outcomes.

The present study suggests that loneliness could be considered as a potential target of intervention in the context of mental health problems. Note that our findings showed the association of loneliness with mental health problems, but not its causal effect. Loneliness can arise for various reasons, leading to the issue of “multiple versions of treatment,” and thereby complicating a unique definition of its causal effect (Hernán & Robins, 2020). Future studies should thus focus on examining the effects of potential interventions targeting loneliness to improve mental health, such as enhancing social support and addressing maladaptive social cognition (Masi, Chen, Hawkley, & Cacioppo, 2011). It is likely that such interventions can be delivered in naturalistic settings (for example, school climate interventions) outside of the formal psychiatric system (Jefferson et al., 2023; Shinde et al., 2018), allowing potential benefits while minimizing stigma and barriers to care. While meta-analytic evidence shows that interventions targeting loneliness can ameliorate loneliness itself (Masi et al., 2011), further research is warranted to examine whether these interventions lead to improvements in mental health problems.

The present study has several limitations. First, although we employed the longitudinal design and rich confounder adjustment, the possibility of residual confounding remains due to the nature of observational studies (Narita & Furukawa, 2025). Second, measurement bias may potentially exist, particularly with self-reported measures. The standardized measures used in the present study were not formally validated in Japanese, which might have further contributed to measurement bias. Loneliness was assessed using a single-item measure, which may not fully capture the multidimensional nature of the construct. Single-item assessments are also vulnerable to random measurement error and social desirability bias (Mund et al., 2023), potentially limiting the robustness and interpretability of the findings. Nonetheless, the prevalence of loneliness in our cohort (14.6% at age 12 and 9.8% at age 14) was broadly consistent with international estimates for adolescents (9.2%–14.4%) (Surkalim et al., 2022), although differences in cultural norms and social contexts that shape the reporting and interpretation of loneliness should be considered when making cross-national comparisons. Third, we lacked data on potentially important confounders, such as physical injuries (Stickley et al., 2020; Stickley et al., 2019). We used physical punishment as a proxy for adverse childhood experiences (Stickley et al., 2020, 2021) but did not consider other stressful or traumatic exposures. Genetic factors may serve as important confounders, while such factors may also serve as effect modifiers (Andreu-Bernabeu et al., 2025). Fourth, for severity outcomes, we summarized associations using coefficients that represent differences in raw scale scores; however, minimally important differences for these measures have not been clearly established for Japanese adolescents, making it difficult to determine whether these coefficients are clinically meaningful on their own. By contrast, the absolute risk increases shown by the RDs for binary outcomes may be more clinically interpretable with respect to the presence of mental health problems. Lastly, our sample, predominantly of Asian ethnicity from a highly urbanized city, might not represent rural or more diverse racial or geographic contexts; however, it also allows consideration of loneliness and mental health in a non-Western setting, where cultural and social factors relevant to these constructs may differ.

## Conclusions

This study contributes to the evidence on adolescents by examining the association of loneliness patterns across time with subsequent psychotic experiences and other mental health problems. The findings suggest that remission of loneliness is associated with reduced risk and severity of psychotic experiences, highlighting the potential importance of intervening in loneliness in adolescence. Future studies should focus on the effect of interventions targeting loneliness on mental health problems.

## Supplementary Material

Supporting Information

## Figures and Tables

**Figure 3:**
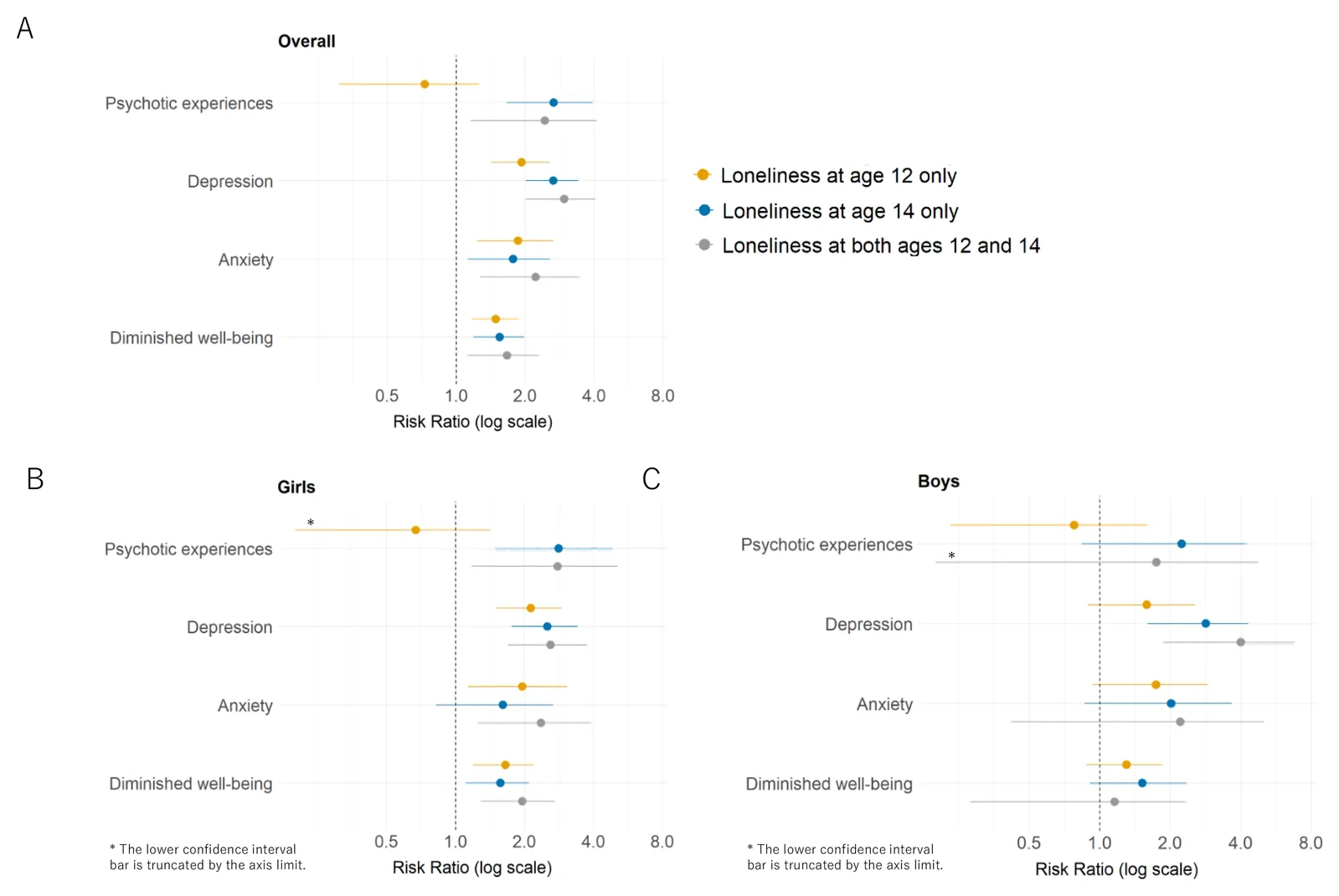
Loneliness patterns (ages 12 and 14) and subsequent risk of mental health problems (age 16) evaluated by the g-formula: Risk ratio in (A) Overall sample, (B) Girls, and (C) Boys.

**Table 1:** Baseline characteristics at age 10 by loneliness status at age 12 (see Table S2 for details of the missing data for each variable).

		Loneliness at age 12		
Variables at age 10	Overall (N = 3,171)	Never (N = 2,171)	Sometimes or always (N = 399)	Missing (N = 651)
Age (months), mean (SD)	122 (3.30)	122 (3.27)	122 (3.39)	122 (3.36)
Gender, n (%)				
Female	1,487 (46.9)	963 (45.4)	222 (55.6)	302 (46.4)
Male	1,684 (53.1)	1,158 (54.6)	177 (44.4)	349 (53.6)
IQ, mean (SD)	108 (14.1)	108 (14.2)	107 (13.3)	107 (14.3)
Body mass index, mean (SD)	16.8 (2.25)	16.8 (2.19)	16.8 (2.51)	16.7 (2.28)
Household income, JPY, n (%)				
< 5,000,000	620 (19.6)	397 (18.7)	87 (21.8)	136 (20.9)
5,000,000 to 9,999,999	1,509 (47.6)	1,037 (48.9)	193 (48.4)	279 (42.9)
≥ 10,000,000	917 (28.9)	618 (29.1)	102 (25.6)	197 (30.3)
Living arrangement, n (%)				
With father	2,847 (89.8)	1,914 (90.2)	363 (91.0)	570 (87.6)
Separated from father	312 (9.8)	200 (9.4)	35 (8.8)	77 (11.8)
Neighborhood characteristics, mean (SD)	12.7 (3.17)	12.7 (3.22)	12.8 (2.97)	12.6 (3.10)
Physical punishment, n (%)				
Never or rarely	1,844 (58.2)	1,253 (59.1)	226 (56.6)	365 (56.1)
Sometimes, often, or always	1,314 (41.4)	860 (40.5)	171 (42.9)	283 (43.5)
Problematic internet use, mean (SD)	2.74 (3.46)	2.65 (3.38)	3.18 (3.58)	2.80 (3.64)
Loneliness, n (%)				
Never	2451 (77.3)	1740 (82.0)	224 (56.1)	487 (74.8)
Sometimes or always	685 (21.6)	360 (17.0)	171 (42.9)	154 (23.7)
Psychotic experiences, mean (SD)	1.13 (1.20)	1.13 (1.19)	1.11 (1.24)	1.12 (1.22)
Depressive symptoms, mean (SD)	4.76 (4.58)	4.24 (4.17)	7.18 (5.45)	4.93 (4.74)
Anxiety symptoms, mean (SD)	2.97 (3.03)	2.81 (2.88)	3.73 (3.55)	3.03 (3.10)
Well-being, mean (SD)	79.0 (16.5)	80.2 (15.9)	74.7 (17.9)	77.9 (16.8)

SD, standard deviation; NA, not applicable

**Table 2:** Association between loneliness patterns across time (ages 12 and 14) and the severity of mental health problems (age 16)

		Outcomes at age 16			
		Psychotic experiences	Depressive symptoms	Anxiety symptoms	Well-being
Loneliness at age 12	Loneliness at age 14	*β* [95% CI]	*β* [95% CI]	*β* [95% CI]	*β* [95% CI]
Overall (N = 3,171)					
Never	Never	0.00 (Reference)	0.00 (Reference)	0.00 (Reference)	0.00 (Reference
Sometimes or always	Never	0.01 [-0.04, 0.08]	1.74 [1.07, 2.43]	0.88 [0.43, 1.37]	-4.86 [-7.83, -1.98]
Never	Sometimes or always	0.26 [0.12, 0.42]	2.62 [1.63, 3.57]	0.64 [0.15, 1.18]	-5.82 [-9.40, -2.35]
Sometimes or always	Sometimes or always	0.28 [0.10, 0.48]	3.62 [2.06, 5.07]	1.00 [0.25, 1.78]	-7.01 [-12.12, -1.68]
Girls (N = 1,487)					
Never	Never	0.00 (Reference)	0.00 (Reference)	0.00 (Reference)	0.00 (Reference)
Sometimes or always	Never	0.01 [-0.06, 0.10]	2.39 [1.39, 3.44]	1.06 [0.32, 1.83]	-5.91 [-10.15, -1.97]
Never	Sometimes or always	0.23 [0.06, 0.42]	2.84 [1.63, 4.22]	0.46 [-0.17, 1.13]	-6.78 [-11.48, -2.34]
Sometimes or always	Sometimes or always	0.32 [0.09, 0.58]	3.71 [1.87, 5.50]	1.08 [0.10, 2.13]	-8.48 [-14.43, -2.51]
Boys (N = 1,684)					
Never	Never	0.01 (Reference)	0.00 (Reference)	0.00 (Reference)	0.00 (Reference)
Sometimes or always	Never	0.01 [-0.07, 0.10]	1.12 [0.26, 2.03]	0.69 [0.12, 1.27]	-3.62 [-7.53, -0.03]
Never	Sometimes or always	0.30 [0.06, 0.60]	2.24 [0.73, 3.87]	0.94 [0.15, 1.82]	-4.40 [-9.86, 1.13]
Sometimes or always	Sometimes or always	0.19 [-0.08, 0.56]	3.45 [1.25, 6.03]	0.84 [-0.48, 2.34]	-4.14 [-14.09, 4.96]

CI, confidence interval Analysis was conducted using the g-formula. Missing data were handled using multiple imputation by chained equations. CIs were derived from the distribution of 4,000 estimates, which resulted from 200 bootstrap samples drawn from each of the 20 imputed datasets. The models adjusted for age, gender, IQ, body mass index, household income, physical punishment, living arrangement, neighborhood cohesion, problematic internet use, loneliness, and each mental health variable measured at age 10 as well as body mass index, physical punishment, problematic internet use, and each mental health variable at age 12.

## Data Availability

The Tokyo Teen Cohort data underlying this article are not publicly available due to participant privacy and ethical restrictions. De-identified data may be available to qualified researchers upon reasonable request and with approval from the TTC data governance committee and relevant ethics boards.

## Key points

### What’s known?

- Adolescent loneliness has typically been assessed only at a single time point in mental health research.

### What’s new?

- Persistent loneliness at ages 12 and 14 was associated with increased risk of mental health problems at age 16.
- No association was found for adolescents whose loneliness had remitted by age 14.

### What’s relevant?

- The risk of mental health problems may not be permanent and could be mitigated if loneliness remits.

## Key points

- Evidence on the association between loneliness and psychotic experiences in adolescents remains limited, and loneliness has typically been assessed at a single time point.
- Using longitudinal data from the Tokyo Teen Cohort, persistent loneliness at ages 12 and 14 was associated with increased risk and greater severity of psychotic experiences, depression, anxiety, and diminished well-being at age 16.
- No association was found for adolescents whose loneliness had remitted by age 14, and findings were generally similar for other mental health problems. Associations were consistent across genders, although the association with well-being appeared particularly important for girls.
- The dynamics of loneliness are linked to adolescent mental health problems. The risk may not be permanent and could be mitigated if loneliness remits. Further research examining interventions that target loneliness is warranted.
