# Supplementary material for: Loneliness patterns across time and subsequent risk of psychotic experiences, depression, anxiety, and diminished well-being in adolescents
Source: J Child Psychol Psychiatry. Author manuscript; Available in PMC 2026 Jul 27. (PMC7619271; doi:10.1111/jcpp.70114)
Supplement: Supporting Information [file EMS215949-supplement-Supporting_Information.docx]

**Method S1** Details on the measurement of covariates.

The model included age (months, continuous), gender (dichotomous), IQ (continuous), body mass index (continuous), household income (JPY, < 5,000,000, 5,000,000 to 9,999,999, or ≥ 10,000,000, categorical), physical punishment (dichotomous, never or rarely vs. sometimes, often, or always), living arrangement (dichotomous, with father vs. separated from father), neighborhood cohesion (continuous), problematic internet use (continuous), and loneliness (dichotomous). Per analysis, we included the baseline level of each mental health condition (continuous), thereby mitigating the possibility of reverse causation. IQ was evaluated using the short form of the Wechsler Intelligence Scale for Children-Third Edition.^1^ Neighborhood cohesion was assessed using the Neighborhood Collective Efficacy scale,^2^ where a higher total score indicates greater neighborhood cohesion (Cronbach’s *α*, 0.87). Problematic internet use was evaluated using the modified version of the Compulsive Internet Use Scale,^3^ where a higher total score indicates greater problematic internet use (Cronbach’s *α*, 0.87).

1. Inada N, Kamio Y. Short forms of the Japanese version WISC-3 for assessment of children with autism spectrum disorders. Jpn J Child Adolesc Psychiatr (Japanese). 2010;51:11-19.

2. Nakanishi M, Yamasaki S, Ando S, et al. Neighborhood Social Cohesion and Dementia-Related Stigma Among Mothers of Adolescents in the Pre- and Current COVID-19 Period: An Observational Study Using Population-Based Cohort Data. J Alzheimers Dis. 2022;88(2):493-502.

3. Meerkerk GJ, Van Den Eijnden RJJM, Vermulst AA, Garretsen HFL. The Compulsive Internet Use Scale (CIUS): some psychometric properties. Cyberpsychol Behav. 2009;12(1):1-6.

**Figure S1** Flowchart of participant recruitment in the Tokyo Teen Cohort.

**
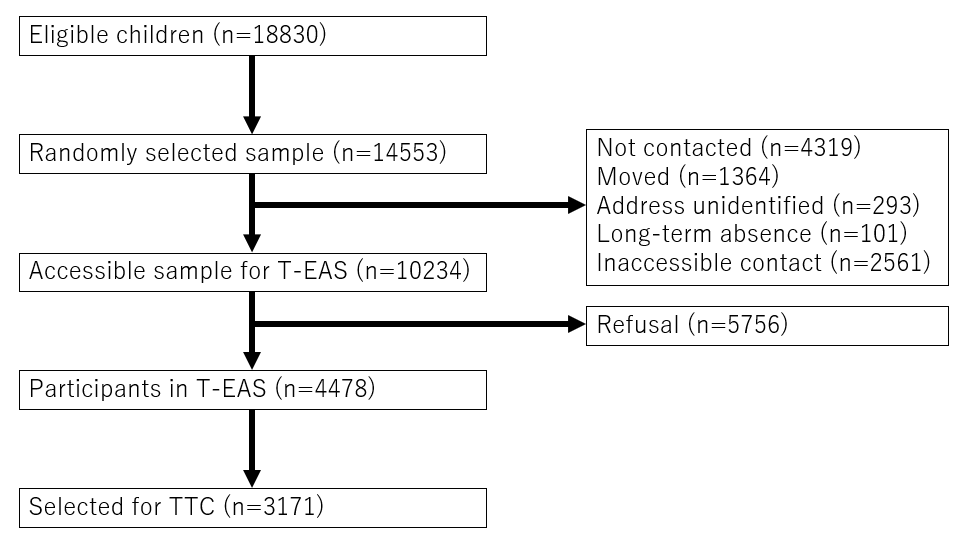
**

T-EAS, Tokyo Early Adolescence Survey; TTC, Tokyo Teen Cohort

**Figure S2** A causal directed acyclic graph showing the associations between loneliness (exposures at ages 12 and 14), covariates (confounders at ages 10 and 12), and mental health problems (outcomes at age 16).

**
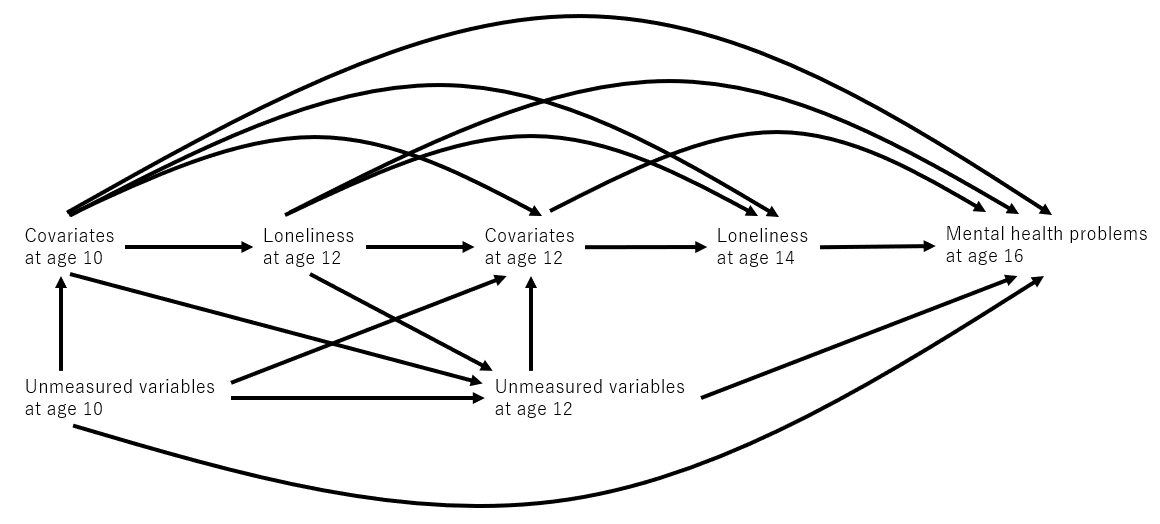
**

**Table S1** The Adolescent Psychotic-like Symptom Screener.

| Questionnaire items | Abbreviation |
| --- | --- |
| 1. Some people believe that their thoughts can be read by another person. Have other people ever read your mind? | Mind reading |
| 2. Have you ever had messages sent just to you through TV or radio? | TV/radio |
| 3. Have you ever thought that people are following or spying on you? | Spying |
| 4. Have you ever heard voices or sounds that no one else can hear? | Auditory hallucinations |
| 5. Have you ever felt you were under the control of some special power? | Controlled |
| 6. Have you ever seen things that other people could not see? | Visual hallucinations |
| 7. Have you ever felt like you had extraspecial powers? | Grandiosity |

**Table S2** Details of missing data, n (%).

|  |  | Loneliness at age 12 | | |
| --- | --- | --- | --- | --- |
| Variables | Overall  (N = 3,171) | Never  (N = 2,171) | Sometimes or always  (N = 399) | Missing  (N = 651) |
| Age (months) at age 10 | 4 (0.1) | 3 (0.1) | 0 (0) | 1 (0.2) |
| Gender | 0 (0) | 0 (0) | 0 (0) | 0 (0) |
| IQ at age 10 | 3 (0.1) | 1 (0.0) | 0 (0) | 2 (0.3) |
| Body mass index at age 10 | 8 (0.3) | 5 (0.2) | 1 (0.3) | 2 (0.3) |
| Household income at age 10 | 125 (3.9) | 69 (3.3) | 17 (4.3) | 39 (6.0) |
| Living arrangement at age 10 | 12 (0.4) | 7 (0.3) | 1 (0.3) | 4 (0.6) |
| Neighborhood characteristics at age 10 | 13 (0.4%) | 8 (0.4%) | 1 (0.3%) | 4 (0.6%) |
| Physical punishment at age 10 | 13 (0.4) | 8 (0.4) | 2 (0.5) | 3 (0.5) |
| Loneliness at age 10 | 35 (1.1) | 21 (1.0) | 4 (1.0) | 10 (1.5) |
| Problematic internet use at age 10 | 69 (2.2) | 40 (1.9) | 10 (2.5) | 19 (2.9) |
| Psychotic experiences at age 10 | 127 (4.0) | 84 (4.0) | 12 (3.0) | 31 (4.8) |
| Depressive symptoms at age 10 | 205 (6.5) | 134 (6.3) | 16 (4.0) | 55 (8.4) |
| Anxiety symptoms at age 10 | 6 (0.2) | 3 (0.1) | 0 (0) | 3 (0.5) |
| Well-being at age 10 | 16 (0.5) | 10 (0.5) | 0 (0) | 6 (0.9) |
| Body mass index at age 12 | 617 (19.5) | 10 (0.5) | 4 (1.0) | 603 (92.6) |
| Problematic internet use at age 12 | 202 (6.4) | 28 (1.3) | 5 (1.3) | 169 (26.0) |
| Physical punishment at age 12 | 425 (13.4) | 11 (0.5) | 6 (1.5) | 408 (62.7) |
| Psychotic experiences at age 12 | 632 (19.9) | 430 (20.3) | 78 (19.5) | 124 (19.0) |
| Depressive symptoms at age 12 | 692 (21.8) | 28 (1.3) | 13 (3.3) | 651 (100) |
| Anxiety symptoms at age 12 | 446 (14.1) | 24 (1.1) | 6 (1.5) | 416 (63.9) |
| Well-being at age 12 | 303 (9.6) | 14 (0.7) | 3 (0.8) | 286 (43.9) |
| Psychotic experiences at age 16 | 1244 (39.2) | 686 (32.3) | 124 (31.1) | 434 (66.7) |
| Depressive symptoms at age 16 | 1251 (39.5) | 692 (32.6) | 126 (31.6) | 433 (66.5) |
| Anxiety symptoms at age 16 | 909 (28.7) | 477 (22.5) | 84 (21.1) | 348 (53.5) |
| Well-being at age 16 | 900 (28.4) | 475 (22.4) | 90 (22.6) | 335 (51.5) |

**Table S3** Association between loneliness patterns (ages 12 and 14) and the presence of mental health problems (age 16)

|  |  | Outcomes at age 16 | |  |  |  |  |  |  |
| --- | --- | --- | --- | --- | --- | --- | --- | --- | --- |
|  |  | Psychotic experiences | | Depression | | Anxiety | | Diminished well-being | |
| Loneliness at age 12 | Loneliness at age 14 | RD, %  [95% CI] | RR  [95% CI] | RD, %  [95% CI] | RR  [95% CI] | RD, %  [95% CI] | RR  [95% CI] | RD, %  [95% CI] | RR  [95% CI] |
| Overall |  |  |  |  |  |  |  |  |  |
| Never | Never | 0.00  (Reference) | 1.00  (Reference) | 0.00  (Reference) | 1.00  (Reference) | 0.00  (Reference) | 1.00  (Reference) | 0.00  (Reference) | 1.00  (Reference) |
| Sometimes or always | Never | -1.3  [-3.6, 1.2] | 0.73  [0.31, 1.26] | 9.8  [4.9, 15.3] | 1.93  [1.43, 2.56] | 5.6  [1.6, 10.0] | 1.86  [1.23, 2.65] | 8.9  [3.2, 15.6] | 1.49  [1.17, 1.89] |
| Never | Sometimes or always | 8.1  [3.4, 13.4] | 2.66  [1.66, 3.94] | 17.5  [11.3, 24.6] | 2.65  [2.01, 3.41] | 5.0  [0.8, 9.7] | 1.77  [1.12, 2.57] | 10.0  [3.6, 17.5] | 1.55  [1.19, 1.98] |
| Sometimes or always | Sometimes or always | 7.1  [0.8, 14.3] | 2.44  [1.16, 4.11] | 20.8  [11.2, 31.3] | 2.96  [2.01, 4.05] | 7.9  [1.9, 15.1] | 2.22  [1.27, 3.46] | 12.3  [2.3, 23.3] | 1.67  [1.12, 2.29] |
| Girls |  |  |  |  |  |  |  |  |  |
| Never | Never | 0.00  (Reference) | 1.00  (Reference) | 0.00  (Reference) | 1.00  (Reference) | 0.00  (Reference) | 1.00  (Reference) | 0.00  (Reference) | 1.00  (Reference) |
| Sometimes or always | Never | -1.6  [-4.5, 1.8] | 0.67  [0.16, 1.42] | 15.1  [7.4, 23.3] | 2.13  [1.51, 2.90] | 7.5  [1.1, 14.5] | 1.96  [1.13, 3.08] | 12.6  [4.0, 21.7] | 1.65  [1.19, 2.19] |
| Never | Sometimes or always | 8.8  [2.7, 16.0] | 2.82  [1.49, 4.83] | 20.4  [11.0, 30.3] | 2.52  [1.76, 3.42] | 4.7  [-1.5, 11.8] | 1.61  [0.82, 2.66] | 11.0  [2.2, 20.1] | 1.57  [1.11, 2.09] |
| Sometimes or always | Sometimes or always | 8.7  [0.9, 17.7] | 2.79  [1.18, 5.09] | 21.6  [9.7, 34.7] | 2.60  [1.70, 3.76] | 10.6  [2.1, 20.7] | 2.36  [1.25, 3.90] | 18.6  [5.9, 31.6] | 1.96  [1.29, 2.71] |
| Boys |  |  |  |  |  |  |  |  |  |
| Never | Never | 0.00  (Reference) | 1.00  (Reference) | 0.00  (Reference) | 1.00  (Reference) | 0.00  (Reference) | 1.00  (Reference) | 0.00  (Reference) | 1.00  (Reference) |
| Sometimes or always | Never | -1.1  [-4.2, 2.6] | 0.78  [0.23, 1.60] | 4.8  [-1.0, 11.3] | 1.59  [0.89, 2.55] | 3.9  [-0.4, 9.0] | 1.74  [0.93, 2.90] | 5.1  [-2.2, 13.5] | 1.30  [0.88, 1.85] |
| Never | Sometimes or always | 6.2  [-0.8, 14.8] | 2.24  [0.84, 4.21] | 14.9  [5.1, 25.3] | 2.84  [1.60, 4.32] | 5.4  [-0.8, 13.0] | 2.02  [0.86, 3.66] | 9.0  [-1.6, 21.6] | 1.52  [0.91, 2.35] |
| Sometimes or always | Sometimes or always | 3.7  [-5.2, 17.4] | 1.75  [1.00e-06, 4.76] | 24.5  [7.4, 44.9] | 4.00  [1.87, 6.78] | 6.4  [-3.2, 20.1] | 2.21  [0.42, 5.03] | 2.8  [-12.7, 22.4] | 1.16  [0.28, 2.34] |

CI, confidence interval

Analysis was conducted using the g-formula.

Missing data were handled using multiple imputation by chained equations.

CIs were derived from the distribution of 4,000 estimates, which resulted from 200 bootstrap samples drawn from each of the 20 imputed datasets.

The models adjusted for age, gender, IQ, body mass index, household income, physical punishment, living arrangement, neighborhood cohesion, problematic internet use, loneliness, and each mental health variable measured at age 10 as well as body mass index, physical punishment, problematic internet use, and each mental health variable at age 12.

**Table S4** Robustness to unmeasured confounding of the observed associations.

| Loneliness at age 12 | Loneliness at age 14 | E-value | Psychotic experiences | Depression | Anxiety | Diminished well-being |
| --- | --- | --- | --- | --- | --- | --- |
| Sometimes or always | Never | Point estimate | 1.00 | 3.27 | 3.12 | 2.34 |
|  |  | Limit of CI | 1.00 | 2.21 | 1.76 | 1.62 |
| Never | Sometimes or always | Point estimate | 4.76 | 4.74 | 2.93 | 2.47 |
|  |  | Limit of CI | 2.71 | 3.43 | 1.49 | 1.67 |
| Sometimes or always | Sometimes or always | Point estimate | 4.31 | 5.37 | 3.87 | 2.72 |
|  |  | Limit of CI | 1.59 | 3.43 | 1.86 | 1.49 |

CI, confidence interval

E-values for point estimates are the minimum strength of association on the risk ratio that unmeasured confounding would need to have above and beyond the adjusted covariates to explain away the estimates.

E-values for limit of CI are the minimum strength of association on the risk ratio that unmeasured confounding would need to have above and beyond the adjusted covariates to shift the 95% CI and include the null value.

**Table S5** Sensitivity analysis for the severity of mental health problems: marginal structural models.

|  |  | Outcomes at age 16 | |  |  |
| --- | --- | --- | --- | --- | --- |
|  |  | Psychotic experiences | Depressive symptoms | Anxiety symptoms | Well-being |
| Loneliness at age 12 | Loneliness at age 14 | *β*  [95% CI] | *β*  [95% CI] | *β*  [95% CI] | *β*  [95% CI] |
| Overall (N = 3,171) |  |  |  |  |  |
| Never | Never |  |  |  |  |
| Sometimes or always | Never | 0.02  [-0.05, 0.09] | 1.86  [1.15, 2.58] | 0.98  [0.47, 1.49] | -5.16  [-8.37, -1.96] |
| Never | Sometimes or always | 0.27  [0.09, 0.45] | 2.59  [1.55, 3.64] | 0.76  [0.16, 1.35] | -5.74  [-9.60, -1.89] |
| Sometimes or always | Sometimes or always | 0.24  [0.01, 0.47] | 3.20  [1.51, 4.89] | 0.56  [-0.39, 1.51] | -5.43  [-11.00, 0.14] |

CI, confidence interval

Missing data were handled using multiple imputation by chained equations.

For each imputed dataset, point estimates and robust (sandwich) variances were obtained; CIs were computed using Rubin’s rules.

The models adjusted for age, gender, IQ, body mass index, household income, physical punishment, living arrangement, neighborhood cohesion, problematic internet use, loneliness, and each mental health variable measured at age 10 as well as body mass index, physical punishment, problematic internet use, and each mental health variable at age 12.

**Table S6** Sensitivity analysis for the incidence of mental health problems: marginal structural models.

|  |  | Outcomes at age 16 | |  |  |
| --- | --- | --- | --- | --- | --- |
|  |  | Psychotic experiences | Depression | Anxiety | Diminished well-being |
| Loneliness at age 12 | Loneliness at age 14 | OR  [95% CI] | OR  [95% CI] | OR  [95% CI] | OR  [95% CI] |
| Overall (N = 3,171) |  |  |  |  |  |
| Never | Never | 1.00  (Reference) | 1.00  (Reference) | 1.00  (Reference) | 1.00  (Reference) |
| Sometimes or always | Never | 0.78  [0.38, 1.61] | 2.33  [1.62, 3.37] | 1.99  [1.26, 3.14] | 1.72  [1.21, 2.45] |
| Never | Sometimes or always | 2.72  [1.66, 4.44] | 3.36  [2.31, 4.89] | 1.79  [1.08, 2.99] | 1.77  [1.21, 2.61] |
| Sometimes or always | Sometimes or always | 2.42  [1.07, 5.49] | 3.70  [1.98, 6.94] | 1.86  [0.87, 3.99] | 1.44  [0.74, 2.80] |

OR, odds ratio, CI, confidence interval

Missing data were handled using multiple imputation by chained equations.

For each imputed dataset, point estimates and robust (sandwich) variances were obtained; CIs were computed using Rubin’s rules.

The models adjusted for age, gender, IQ, body mass index, household income, physical punishment, living arrangement, neighborhood cohesion, problematic internet use, loneliness, and each mental health variable measured at age 10 as well as body mass index, physical punishment, problematic internet use, and each mental health variable at age 12.

**Table S7** Sensitivity analysis: further adjusting for time spent gaming at ages 10 and 12.

|  |  | Outcomes at age 16 | |  |  |
| --- | --- | --- | --- | --- | --- |
|  |  | Psychotic experiences | Depressive symptoms | Anxiety symptoms | Well-being |
| Loneliness at age 12 | Loneliness at age 14 | *β*  [95% CI] | *β*  [95% CI] | *β*  [95% CI] | *β*  [95% CI] |
| Never | Never | 0.00  (Reference) | 0.00  (Reference) | 0.00  (Reference) | 0.00  (Reference) |
| Sometimes or always | Never | 0.01  [-0.04, 0.08] | 1.76  [1.08, 2.46] | 0.88  [0.42, 1.36] | -4.84  [-7.91, -1.93] |
| Never | Sometimes or always | 0.26  [0.12, 0.42] | 2.63  [1.63, 3.56] | 0.65  [0.15, 1.21] | -5.79  [-9.20, -2.20] |
| Sometimes or always | Sometimes or always | 0.28  [0.10, 0.49] | 3.63  [2.17, 5.15] | 0.99  [0.23, 1.78] | -7.01  [-12.21, -1.77] |

CI, confidence interval

Analysis was conducted using the g-formula.

Missing data were handled using multiple imputation by chained equations.

CIs were derived from the distribution of 4,000 estimates, which resulted from 200 bootstrap samples drawn from each of the 20 imputed datasets.

The models adjusted for age, gender, IQ, body mass index, household income, physical punishment, living arrangement, neighborhood cohesion, problematic internet use, time spent gaming, loneliness, and each mental health variable measured at age 10 as well as body mass index, physical punishment, problematic internet use, time spent gaming, and each mental health variable at age 12.

**Table S8** Sensitivity analysis for depressive symptoms: using the Short Mood and Feelings Questionnaire scale, including the loneliness item.

| Loneliness at age 12 | Loneliness at age 14 | *β*  [95% CI] |
| --- | --- | --- |
| Never | Never | 0.00  (Reference) |
| Sometimes or always | Never | 1.76  [1.10, 2.46] |
| Never | Sometimes or always | 2.85  [1.88, 3.81] |
| Sometimes or always | Sometimes or always | 4.08  [2.41, 5.70] |

CI, confidence interval

Analysis was conducted using the g-formula.

Missing data were handled using multiple imputation by chained equations.

CIs were derived from the distribution of 4,000 estimates, which resulted from 200 bootstrap samples drawn from each of the 20 imputed datasets.

The models adjusted for age, gender, IQ, body mass index, household income, physical punishment, living arrangement, neighborhood cohesion, problematic internet use, loneliness, and depressive symptoms measured at age 10 as well as body mass index, physical punishment, problematic internet use, and depressive symptoms at age 12.

**Table S9** Sensitivity analysis: examining loneliness patterns at ages 12 and 16.

|  |  | Outcomes at age 16 | |  |  |
| --- | --- | --- | --- | --- | --- |
|  |  | Psychotic experiences | Depressive symptoms | Anxiety symptoms | Well-being |
| Loneliness at age 12 | Loneliness at age 16 | *β*  [95% CI] | *β*  [95% CI] | *β*  [95% CI] | *β*  [95% CI] |
| Overall (N = 3,171) |  |  |  |  |  |
| Never | Never |  |  |  |  |
| Sometimes or always | Never | 0.02  [-0.04, 0.08] | 1.06  [0.53, 1.63] | 0.84  [0.36, 1.33] | -3.79  [-6.44, -1.02] |
| Never | Sometimes or always | 0.32  [0.21, 0.44] | 6.72  [5.80, 7.63] | 0.91  [0.51, 1.35] | -11.54  [-14.50, -8.65] |
| Sometimes or always | Sometimes or always | 0.29  [0.14, 0.46] | 7.76  [6.49, 8.98] | 1.34  [0.62, 2.10] | -13.61  [-17.87, -9.29] |

CI, confidence interval

Missing data were handled using multiple imputation by chained equations.

For each imputed dataset, point estimates and robust (sandwich) variances were obtained; CIs were computed using Rubin’s rules.

The models adjusted for age, gender, IQ, body mass index, household income, physical punishment, living arrangement, neighborhood cohesion, problematic internet use, loneliness, and each mental health variable measured at age 10 as well as body mass index, physical punishment, problematic internet use, and each mental health variable at age 12.
